# The clinical efficacy of Shenmai injection in the prophylaxis and treatment of intradialytic hypotension: A protocol for systematic review and meta-analysis

**DOI:** 10.1097/MD.0000000000030949

**Published:** 2022-10-14

**Authors:** Zhen Xiang, Xin Lin, Jun Wang, Guodan Yu

**Affiliations:** a Department of Nephropathy Rheumatism, The Central Hospital of Enshi Tujia and Miao Autonomous Prefecture, Enshi, Hubei Province, China.

**Keywords:** intradialytic hypotension, meta-analysis, protocol, Shenmai injection

## Abstract

Intradialytic hypotension (IDH) is a common complication in hemodialysis. IDH can induce vomiting, chest tightness and syncope, and hemodialysis shall be discontinued in patients with severe IDH. As is revealed in related studies, Shenmai injection (SMI) can be used in the prophylaxis and treatment of IDH. However, there is still a lack of consensus about the efficacy among reported studies, which cannot provide compelling evidence. Therefore, a meta-analysis was conducted in this study to further investigate the efficacy and safety of SMI in the prophylaxis and treatment of IDH.

**Methods:**

PubMed, Web of Science, Scopus, Cochrane Library, Embase, China Scientific Journal Database, China National Knowledge Infrastructure, Chinese Biomedical Literature Database, and Wanfang Data were systematically retrieved from their establishment to June 2022. Subsequently, literature screening, data extraction, quality evaluation and cross-checking of results were performed according to the Cochrane Handbook. Besides, a meta-analysis was performed with the assistance of Revman 5.3 software.

**Results:**

This study will evaluate whether SMI is effective in the prophylaxis and treatment of IDH.

**Conclusions:**

The latest evidence for the efficacy and safety of SMI in the prevention and treatment of IDH can be provided through this study.

## 1. Introduction

Intradialytic hypotension (IDH) is one of the common clinical complications in patients receiving maintenance hemodialysis, with a high incidence of about 20% to 30%.^[1–3]^ IDH can be induced by multiple factors, mainly including the imbalance of the ultrafiltration rate and capillary refill rate, as well as the decrease in cardiac function, electrolyte disturbance, high dialysate temperature, and immune response to hemodialysis.^[4]^ This disease often occurs in the middle and late stages of hemodialysis. Patients mainly present with nausea, vomiting, palpitation, sweating and paleness, and hypotensive shock and sudden death may occur in severe cases.^[5,6]^ Therefore, it is of great significance to conduct early prophylaxis and timely management of IDH is important.

Shenmai injection (SMI) is a compound of effective saponins extracted from Ginseng (*Panax ginseng*) and Radix Ophiopogonis (*Ophiopogon japonicus*).^[7]^ This formula originates from *Qianjin Pharmacopoeia* and has the effects of rescuing rebellion and returning Yang, consolidating deficiency, nourishing Yin and clearing the heart, restoring the pulse and benefiting Qi, and benefiting Qi and generating fluid.^[8,9]^ It is mainly employed in the treatment of “deficiency labor” and “syncope” in clinical practice.^[10]^ According to modern pharmacological research, ginsenosides, maitake saponins and maitake flavonoids in Ginseng and Radix Ophiopogonis can improve the adaptability of myocardial cells to hypoxia, enhance myocardial metabolism, protect the lipid structure of myocardial cell membranes, stabilize cell membranes, promote the synthesis and release of prostaglandins from myocardial cells, and regulate vascular endothelial function.^[7,11–13]^ It has been found that giving an appropriate amount of SMI to patients receiving hemodialysis can effectively reduce the incidence of IDH.

As is revealed in many randomized controlled trials,^[14–16]^ SMI could achieve significant efficacy in the prophylaxis and treatment of IDH. However, there are some limitations due to varying effect sizes and small sample sizes, as well as a lack of relevant systematic evaluation and conclusions. Based on that, the efficacy and safety of SMI in the prophylaxis and treatment of IDH were evaluated by a meta-analysis, with the aim of providing a basis for its clinical application.

## 2. Methods

### 2.1. Study registration

The protocol of this review was registered in PROSPERO (CRD42022328480). Meanwhile, it was reported as per the statement guidelines of preferred reporting items for the systematic review and meta-analysis protocol.^[17]^

### 2.2. Inclusion criteria

#### 2.2.1. Types of this study

In this study, randomized controlled trials would be conducted to investigate the efficacy and safety of SMI in the prophylaxis and treatment of IDH.

#### 2.2.2. Types of participants

1. The diagnostic criteria of Kidney Disease Outcomes Quality Initiative (KDOQI) were adopted during the inclusion process. KDOQI guidelines for hemodialysis hypotension diagnosis, that is, a decrease in mean arterial pressure (MAP) ≥10 mm Hg or a decrease in systolic blood pressure (SBP) ≥20 mm Hg, were adopted during the inclusion process, which may be accompanied by the symptoms of hypotension, such as dizziness, fatigue and weakness;
2. Patients with an age ≥18 years;
3. Patients with a regular hemodialysis period >90 days;
4. Patients with the hemodialysis frequency being 3 times a week.

#### 2.2.3. Types of interventions

In the experimental group, patients were treated with SMI, regardless of the dose, timing and duration of administration, or combined with conventional treatment. In the control group, patients were treated with conventional treatment alone, including reducing blood flow rate, controlling ultrafiltration, and applying vasoactive drugs.

#### 2.2.4. Types of outcome indexes

##### 2.2.4.1. Primary outcomes

The incidence of IDH was the primary outcome.

##### 2.2.4.2. Secondary outcomes

The secondary outcomes were composed of the total effective rate, the mean arterial pressure after hemodialysis and adverse events.

### 2.3. Exclusion criteria

1. Non-randomized controlled trials (studies related to animal experiments, theoretical discussion, review and experience summary, case reports, analytical articles and so forth);
2. Duplicate publications;
3. Articles without complete outcome indicators.

### 2.4. Information sources and retrieval strategy

The foreign and domestic articles on the prophylaxis and treatment of IDH with SMI were retrieved from PubMed, Web of Science, Scopus, Cochrane Library, Embase, China Scientific Journal Database, China National Knowledge Infrastructure, Chinese Biomedical Literature Database, and Wanfang Data. Besides, relevant articles were also obtained from the Internet as supplements. In addition, the references included in the article were also be consulted, and other relevant articles were manually searched to obtain relevant information that was not found during the above retrieval. All retrieval strategies were determined after multiple searches. The retrieval period was determined from the establishment of the database to June 2022. The research strategy for PubMed is shown in Table 1.

**Table 1:** Search strategy in PubMed database.

Number	Search terms
#1	Dialysis [MeSH]
#2	Dialyses [Title/Abstract]
#3	Renal dialysis [MeSH]
#4	Dialysis, extracorporeal [Title/Abstract]
#5	Dialysis, renal [Title/Abstract]
#6	Extracorporeal dialysis [Title/Abstract]
#7	Hemodialysis [Title/Abstract]
#8	Dialyses, extracorporeal [Title/Abstract]
#9	Dialyses, renal [Title/Abstract]
#10	Extracorporeal dialyses [Title/Abstract]
#11	Hemodialyses [Title/Abstract]
#12	Renal dialyses [Title/Abstract]
#13	or/1–12
#14	Shenmai injection [Title/Abstract]
#15	Shenmai [Title/Abstract]
#16	or/14–15
#17	Randomized controlled trials as topic [MeSH]
#18	Clinical trials, randomized [Title/Abstract]
#19	Controlled clinical trials, randomized [Title/Abstract]
#20	Trials, randomized clinical [Title/Abstract]
#21	Random* [Title/Abstract]
#22	or/17–21
#23	#13 and #16 and #22

### 2.5. Data collection and analysis

#### 2.5.1. Data extraction and management

**Figure 1.**
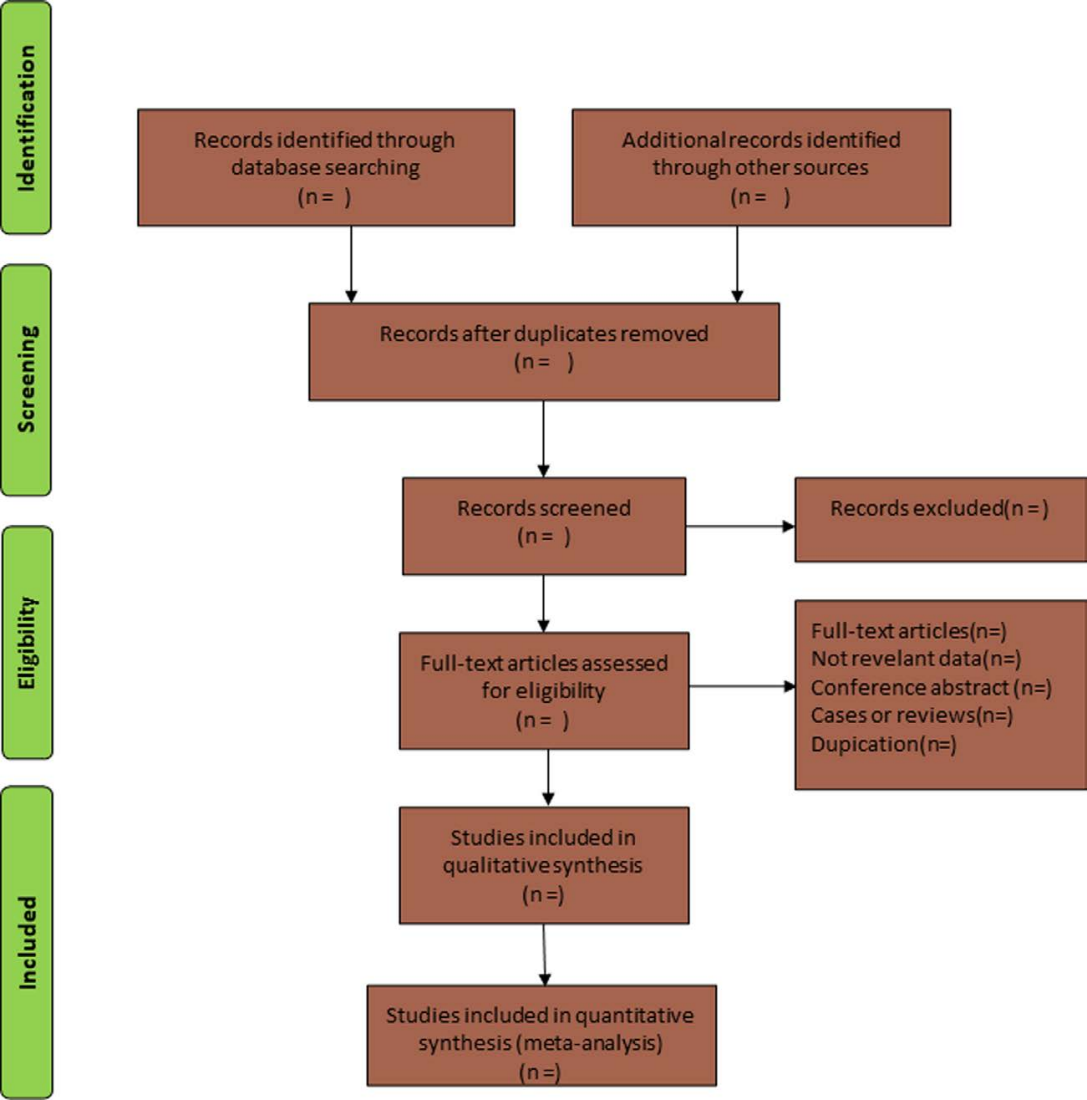
Flow diagram of study selection process.

The acquired articles were independently screened and cross-checked by two investigators according to the inclusion and exclusion criteria. The title of the article was read first. If it was in line with the inclusion criteria, the abstract and full text were further read and included if they were consistent with the inclusion criteria. In case of disagreement, a third researcher was consulted to assist in the determination or experts were consulted to resolve it. The data extraction table was prepared by two researchers independently based on the method recommended by the Cochrane Collaboration. The contents mainly included: basic information of the study, such as the first author of the included article and date of publication; types of study design, interventions, and controls; clinical indicators, including the incidence of IDH, the total effective rate, and the mean arterial pressure after hemodialysis. The specific screening process is shown in Figure 1.

#### 2.5.2. Assessment of risk of bias

The risk of bias in the included articles was assessed according to the Risk of Bias Assessment Tool in *Cochrane Handbook of Systematic Reviews* (5th edition).^[18]^ Two investigators evaluated the risk of bias for each item as unclear, low risk, and high risk of bias. In case of disagreement, the third investigator was responsible for resolving it.

#### 2.5.3. Measures of treatment effects

For dichotomous outcomes, the risk ratio was used in the meta-analysis. The continuous data were expressed as the standardized mean difference. All of these data were summarized with a 95% confidence interval.

#### 2.5.4. Management of missing data

If there are missing data in the included articles, the authors would be contacted by email to obtain relevant information. If the complete data cannot be obtained, it would be recorded in the bias risk assessment. Meanwhile, data analysis would be performed based on the existing data.

#### 2.5.5. Assessment of heterogeneity and data synthesis

A meta-analysis was performed with the assistance of RevMan 5.3 software (The Cochrane Collaboration, Oxford, UK). The included data were tested in terms of the heterogeneity with I2 <50% indicates a good homogeneity of the study, and a fixed-effects model would be used to conduct a meta-analysis. I2 >50% indicates a larger heterogeneity of the study, and the source of heterogeneity would be explored. If the significant clinical heterogeneity and the source of heterogeneity cannot be found, a random-effects model would be selected for analysis.

#### 2.5.6. Assessment of reporting biases

A funnel plot was utilized to test the potential publication bias.^[19]^

#### 2.5.7. Subgroup analysis

A subgroup analysis was conducted based on the primary intervention in the control group.

#### 2.5.8. Sensitivity analysis

A sensitivity analysis was conducted with the fixed-effects and random-effects models, in an attempt to test the stability of the results.

#### 2.5.9. Ethics and dissemination

Ethical approval is not required for this protocol due to the fact that the systematic evaluation is not involved with the data of individual patients. The results will be published in peer-reviewed journals and presented at related conferences.

## 3. Discussion

SMI is commonly used in the prophylaxis and treatment of IDH owing to its functions of benefiting Qi and strengthening the heart, generating fluid and restoring the blood vessels, activating blood circulation and removing blood stasis, and strengthening healthy qi to eliminate pathogens.^[11,20–22]^ However, there is no meta-analysis on the prophylaxis and treatment of IDH with SMI at home and abroad. In this study, the incidence of hemodialysis-related hypotension, the total effective rate and the mean arterial pressure after hemodialysis were analyzed based on the results of clinical studies on the prophylaxis and treatment of IDH with SMI. These findings could provide the evidence-based medical basis for the application of SMI in the prophylaxis and treatment of IDH.

## Author contributions

**Conceptualization:** Zhen Xiang, Guodan Yu.

**Data curation:** Zhen Xiang, Xin Lin.

**Funding acquisition:** Guodan Yu.

**Methodology:** Xin Lin.

**Resources:** Xin Lin, Jun Wang.

**Supervision:** Guodan Yu.

**Validation:** Jun Wang.

**Visualization:** Jun Wang.

**Writing – original draft:** Zhen Xiang, Guodan Yu.

**Writing – review & editing:** Zhen Xiang, Guodan Yu.
